# Magnetic Porous Hydrogel-Enhanced Wearable Patch Sensor for Sweat Zinc Ion Monitoring

**DOI:** 10.3390/s24175627

**Published:** 2024-08-30

**Authors:** Yao Chu, Zhengzhong LvZeng, Kaijie Lu, Yangyang Chen, Yichuan Shen, Kejia Jing, Haifeng Yang, Wanxin Tang

**Affiliations:** College of Chemistry and Materials Science, Shanghai Normal University, Shanghai 200234, China

**Keywords:** magnetic porous hydrogel, uniform distribution, natural sweat detection, fast sweat collection, sweat trace metals

## Abstract

Wearable sensors for sweat trace metal monitoring have the challenges of effective sweat collection and the real-time recording of detection signals. The existing detection technologies are implemented by generating enough sweat through exercise, which makes detecting trace metals in sweat cumbersome. Generally, it takes around 20 min to obtain enough sweat, resulting in dallied and prolonged detection signals that cannot reflect the endogenous fluctuations of the body. To solve these problems, we prepared a multifunctional hydrogel as an electrolyte and combined it with a flexible patch electrode to realize real-time monitoring of sweat Zn^2+^. Such hydrogel has magnetic and porous properties, and the porous structure of hydrogel enables a fast absorption of sweat, and the magnetic property of the addition of fabricated Fe_3_O_4_ NPs not only improves the conductivity but also ensures the adjustable internal structures of the hydrogel. Such a sensing platform for sweat Zn^2+^ monitoring shows a satisfied linear relationship in the concentration range of 0.16–16 µg/mL via differential pulsed anodic striping voltammetry (DPASV) and successfully detects the sweat Zn^2+^ of four volunteers during exercise and resting, displaying a promising path for commercial application.

## 1. Introduction

Wearable sensors have broken out as an emerging technology in health monitoring and personal physiological management. These sensors could provide monitoring data in a non-invasive and continuous way, making them an invaluable tool for medical professionals and individuals [1,2,3]. Sweat is one of the parameters for monitoring body health, and wearable sensors for sweat analysis are particularly significant as they contain information about the body’s metabolic indicators [4,5]. For instance, wearable sensors for detecting trace metals in sweat can reflect the health status of the body [6], since it is easy to see the loss of trace elements through excessive exercise and the diet. Therefore, it is crucial to promote the development of wearable trace metal detection in sweat.

Zinc (Zn) is an essential trace element for life [7], which is required for most biological activities and processes in the human body, and is indispensable for the normal function of many enzymes, transcription factors, and replication factors. Chronic zinc deficiency can lead to hair loss, enteropathy dermatitis, and susceptibility to infections [8]. Even mild zinc deficiency can lead to clinically relevant symptoms that reflect multiple levels of organ dysfunction. When zinc deficiency is severe, both innate and adaptive immunity are severely suppressed, and patients may develop problems such as bullous dermatitis, diarrhea, hair loss, and intellectual disability [9]. Zn in normal human sweat ranges from 0.39 to 1.56 μg/mL, reflecting physiological levels [8,10], and examining sweat for trace metals could support the studies of toxicological and therapeutic aims. Therefore, sensitive analytical methods need to be developed to detect zinc ions in sweat.

Up to now, a few studies have been conducted on wearable sensors detecting sweat Zn^2+^, including the wearable temporary tattoo sensor for the real-time monitoring of Zn^2+^, displaying the acceptable limit of detection (LOD) for sweat Zn^2+^ detection [11]. The fabricated wearable microsensor array for simultaneous heavy metal monitoring in sweat shows an accurate and sensitive sensing platform with a temperature correction system [12]. However, those sensing platforms using long-term exercise for sweat collection to monitor the sweating of trace metals have the challenges of (a) long-term sweat collection prolonging the sensing of trace metals, where the real-time changes in sweat Zn^2+^ cannot be reflected, and (b), how, typically, the sweat-secretion process also involves the evaporation of sweat, leading to the accumulation of trace metals on the sensing surface and an inaccurate detection result. Therefore, it is necessary to build a sweat trace metal sensing platform with a fast and easy-going sweat collection system.

**Figure 1:**
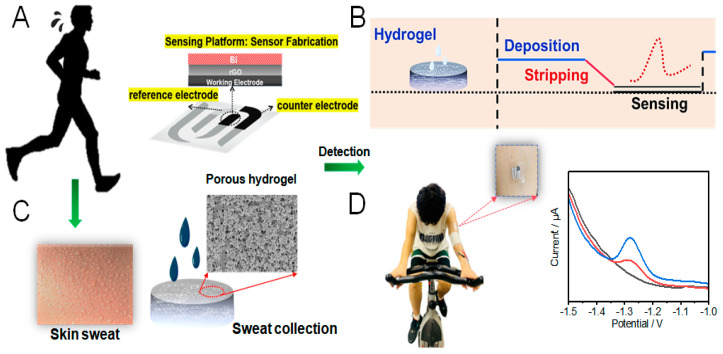
(**A**) A schematic illustration of a flexible patch sensor with multifunctional hydrogel for the sensing of zinc ions (Zn^2+^) in sweat. (**B**) The electrochemical sensing procedure of sweat Zn^2+^ by DPASV. (**C**) Multifunctional hydrogel with a porous construction as a sweat reservoir. (**D**) Real−time monitoring of sweat Zn^2+^ during exercise with the flexible patch sensor stuck on a subject’s forearm.

Herein, we introduce, for the first time, a multifunctional hydrogel with a porous, magnetically regulated structure and good conductivity introduced to the flexible patch sensing platform for detecting sweat Zn^2+^. In Figure 1C, the sensing platform took advantage of the high porosity and capillary effect in a hydrogel that could achieve fast sweat collection [13,14], and the authenticity of the detection results were obtained. Compared with the reported work on sweat Zn^2+^ monitoring, our proposed sensing platform has a short sweating time and a favorable detection limit, reflecting our priority to make a user-friendly wearable patch sensor (Table S1). Additionally, as shown in Figure 1A,B, the typical electrochemical stripping voltametric determination of Zn^2+^ was performed, including the reduced Graphene Oxide (rGO) and bismuth (Bi) film-modified flexible patch electrodes for metal measurements [15,16,17]. The flexible electrode is prepared by the modification of a flexible polyurethane (PU) film as the substrate, showing good compactness with the printing stock [18]. As shown in Figure 1D, the flexible modified electrode patch was attached to the volunteer’s forearm to monitor Zn^2+^, and the porous and easily immobilized magnetic hydrogel could build a compatible sensing surface between the flexible electrode and the skin, making it user-friendly for monitoring Zn^2+^, and achieving a good linearity in the range of 0.16~16 µg/mL. On-body electrochemical measurements of sweat Zn^2+^ during routine activities (cycling and resting) on four recruited subjects are thus demonstrated by implementing our hydrogel-based flexible patch sensor stuck onto human skin.

## 2. Materials and Methods

### 2.1. Materials

Agarose (Adamas-bate), GO (4 mg/mL, Carmery, Huzhou, China), aminated multiwall carbon nanotubes (NH_2_-MWCNT, ~50 µm, Aladdin, Shanghai, China), and Bi standard solution (Xiamen Standards Ltd., Xiamen, China) were used. CaCO_3_ (600 mesh) were purchased from (Junhui Polymer Guangxi Technology Co., Ltd., Hezhou, China). FeCl_2_·4H_2_O (Hengxing Chemical Reagent Co., Ltd., Tianjin, China), MgSO_4_, PbC_4_H_6_O_4_**·**3H_2_O (General-Reagent, Shanghai, China), and CdCl_2_ were purchased from Titan technology Co., Ltd. (Shanghai, China). CuSO_4_**·**5H_2_O and Zn(NO_3_)_2_**·**6H_2_O were purchased from (Sinopharm Group Chemical Reagent Co., Ltd., Shanghai, China). Acetic acid (Jiangsu Qiangsheng Functional Chemical Co., Ltd., Changshu, China) was used. All the reagents were of analytical reagent grade. Elastic waterborne polyurethane (PU) film made of medical adhesive (HUAWEI breathable WPU film, Wuhan, China), polyethylene terephthalate (PET) (Dongsheng plastic material, Shenzhen, China), carbon ink (Inman Graphene Technology Jiangsu Co., Ltd., Huaian, China), silver ink (Shanghai Julong Electronic Technology Co., Ltd., Shanghai, China), and conductive fiber (Shenzhen Huizheng New Material Co., Ltd., Shenzhen, China) were also used.

### 2.2. Instruments

The electrochemical characterizations, including CV, DPASV, and impedance, were performed by using EmStat3 Blue (PalmSens, Houten, The Netherlands) and the CHI600E electrochemical station (CH Instruments, Inc., Shanghai, China). EIS was conducted at a frequency range of 0.01 Hz~100 kHz in 0.1 M KCl containing 5 mmol/L [Fe (CN)_6_]^3−/4−^ solution. SEM images were collected on a JEM-2100F transmission electron microscope (200 kV accelerating voltage, Tokyo, Japan) with an energy dispersive spectrometer.

### 2.3. Preparation of the Fe_3_O_4_ Nanoparticles

Synthesis of iron oxide nanoparticles (Fe_3_O_4_ NPs): In accordance with a method in the previous literature [19], Fe_3_O_4_ NPs were synthesized. In detail, 2.7 g ferric chloride hexahydrate (FeCl_3_·6H_2_O) was dissolved in 80mL glycol (EG) solution and dispersed by ultrasound to form a yellow solution. In addtion, 2.0 g polyethylene glycol (PEG) and 7.2 g anhydrous sodium acetate (NaAc) were added to the mixture, and the solution quickly turned brown under intense agitation. After 30 min, the solution was transferred to a 100 mL sealed polytetrafluoroethylene-lined stainless-steel autoclave and reacted at 200 °C for 8 h. After the end of the reaction, it was taken out after natural cooling, and washed with water and ethanol in turn to clarify the top liquid. It was then dried in the oven at 60 °C and ground in a mortar to obtain the desired Fe_3_O_4_ NPs.

### 2.4. Preparation of Multifunctional Hydrogels

The fabrication process of magnetic porous hydrogel was described as reported in our previous work [18]. Similarly, the agarose was used as a hydrogel template and the CaCO_3_ (~23 µm) as porogen. Agarose and CaCO_3_ were first dissolved in DI water, with stirring and homogeneous mixing. Meanwhile, the NH_2_-MWCNT and Fe_3_O_4_ NPs (~100 nm) in a weight ratio of (1:1) were mixed and added to the mixture of agarose and CaCO_3_, heating and stirring for 10 min at 95 °C. Then, the final mixture was added to a three-dimensional (3D) printed model (1 mm × 1 cm) to cure and achieve the uniformly sized hydrogels. During the curing process, a magnet is used to regulate the inner structures of agarose [20]. In detail, the magnet was placed above the hydrogel-coated 3D-printed model, and the cross-linked structure inside the hydrogel is evenly distributed through the action of the external magnetic field on Fe_3_O_4_ NPs inside the hydrogel (Figure S2). Finally, the hydrochloric acid (HCl) solution was added to the fabricated hydrogels to obtain the porous structures of hydrogels [21].

### 2.5. Preparation of Electrochemical Sensing Platform

The flexible patch electrode was fabricated by using screen-printed technology, including the “SHNU” logo shape-based printed stencil designed by “Adobe Illustrator CS6” software. Commercial carbon ink and silver ink were used to print the three-electrode system. PU film was used as a flexible substrate with a printed working area, reference area, and counter area. In detail, the commercial silver ink was printed on a PU substrate through the designed stencil to define the reference electrode and connection area. Then, the printed electrodes were baked in the oven for 20 min at 80 °C, the carbon ink was printed on the working area and counting area, similarly, baking for 10 min at 80 °C. The proposed flexible patch electrode shows good resistance to mechanical deformation and good skin compactness at different statuses including bending and twisting (Figure S1). 

The fabrication of the flexible electrochemical path sensing platform for the monitoring of sweat Zn^2+^ is displayed in Figure 1A. An amount of 4 mg/mL GO solution was used for the preparation of rGO at −1.2 V for 400 s on the working electrode, after cleaning up the residual GO with DI water, followed by the deposition of Bi at −0.8 V for 400 s, washing twice, and achieving the final flexible electrochemical sensing platform for sweat Zn^2+^.

### 2.6. On-Body Test of Sweat Zn^2+^ by Hydrogel-Based Flexible Patch Sensor

A total of 4 healthy volunteers (1 female and 3 males) were recruited. The flexible Zn patch sensor was stuck to a subject’s forearm to monitor the Zn^2+^ concentration during exercising and resting (Figure 1D). Subjects were asked to ride a stationary cycle and sit on a chair for 8 min, respectively. Meanwhile, during the exercise and rest, along with the DPASV electrochemical method to detect sweat Zn^2+^, all the sensing signals were recorded by an EmStat3 Blue. The DPASV parameters included the following: a pre-deposition voltage set at −1.4 V, pre-deposition tine of 8 min, and a voltage range applied from detection at −1.5 to −0.8 V vs. Ag/AgCl.

## 3. Results and Discussion

### 3.1. Uniform Structural Analysis of Magnetic Porous Hydrogel

**Figure 2:**
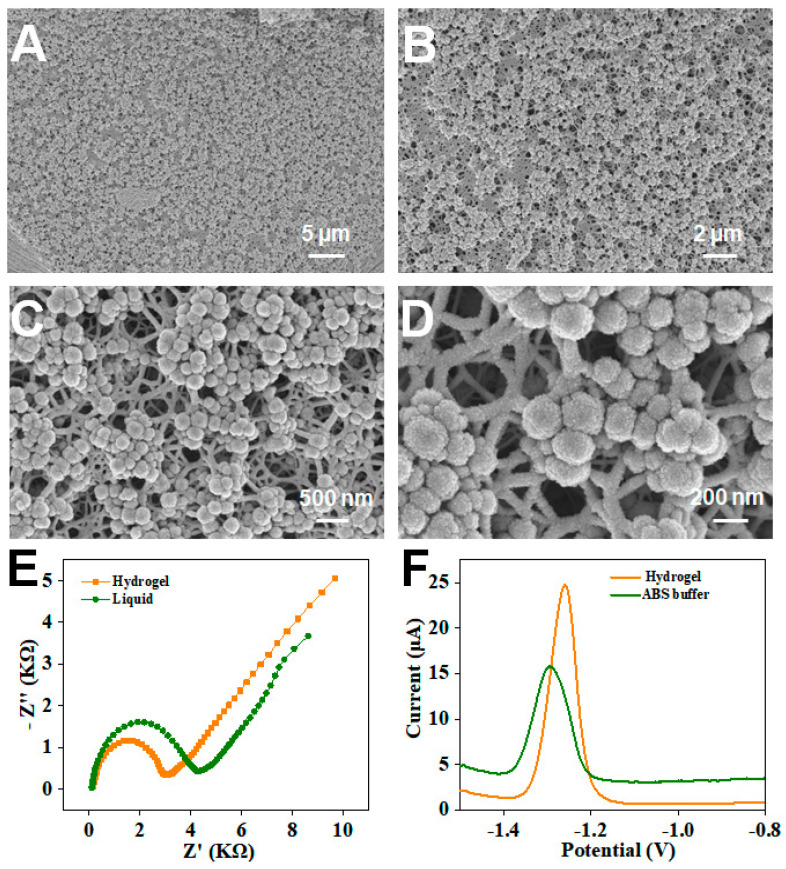
The scanning electron microscope (SEM) images of multifunctional hydrogel. (**A**,**B**) show the different magnifications of the cross−section of the magnetic porous hydrogel. (**C**) The cross-linked network structure of the hydrogel. (**D**) The uniform Fe_3_O_4_ nanoparticles distributed in a hydrogel network structure. (**E**) The impedance of multifunctional hydrogel soaked in 5 mmol/L Ferro/ferricyanide and 0.1 mol/L KCl solution (impedance solution) and impedance solution. (**F**) The electrochemical detection of 1 μg/mL Zn^2+^ by DPASV in acetate buffer (ABS buffer, pH 4.5)-soaked multifunctional hydrogel and ABS buffer, respectively.

The structure of magnetic porous hydrogels was investigated by Scanning Electron Microscopy (SEM). Figure 2A–D show SEM images of the magnetic porous hydrogel in the case of the acidic removal of CaCO_3_ particles, In Figure 2A, it can be seen from the SEM images of the cross-section of part of the gel that a large number of Fe_3_O_4_ NPs are evenly distributed in the hydrogel. Additionally, in Figure 2B, the pores on the surfaces along with the Fe_3_O_4_ NPs of the magnetic porous hydrogels are visible. The magnetic porous hydrogel, with the magnetically regulated structure formed under external magnetic induction, has evenly distributed pores on the surface. Figure 2C,D show the internal structure of magnetic porous hydrogel at magnification; after the magnetic regulation, the internal structure of the hydrogel was distributed in a network, cross-linking and arranged layer by layer; meanwhile, the Fe_3_O_4_ NPs were evenly distributed in network structures, leading to the good conductivity of the hydrogel.

In addition, the electrochemical behavior of such hydrogel was investigated and compared with that of liquid electrolytes on electrodes. Electrochemical impedance spectroscopy (EIS) was used to evaluate the electrochemical properties of multifunctional hydrogels, including for ion exchange and charge transfer. The magnetic porous hydrogel was attached to the flexible electrode for electrochemical tests. EIS curves for magnetic porous hydrogels and electrolyte solution in the frequency range of 1 Hz to 100 kHz, acquired by soaking in a 0.1 M KCl solution containing 5 mmol/L [Fe (CN)_6_]^3−/4−^ are displayed in Figure 2E. Low-frequency regions of the Nyquist plots reflect the diffusion defect of electrolyte ions in hydrogels [22]. The diffusion resistance of magnetic porous hydrogels of Figure 2E showed a relative reduction compared to the EIS results recorded on liquid electrolyte, demonstrating the fabricated magnetic porous hydrogel has good electron transfer properties and conductivity. Meanwhile, the feasibility of the rGO/Bi sensing system for detecting Zn^2+^ was further investigated in Figure 2F. From the comparison between hydrogel and the ABS (4.5) buffer-built sensing system, the hydrogel showed an increased sensing signal for Zn^2+^ at the concentration of 1 μg/mL when measured by DPASV.

### 3.2. Optimization of rGO/Bi Film Sensing Platform

**Figure 3:**
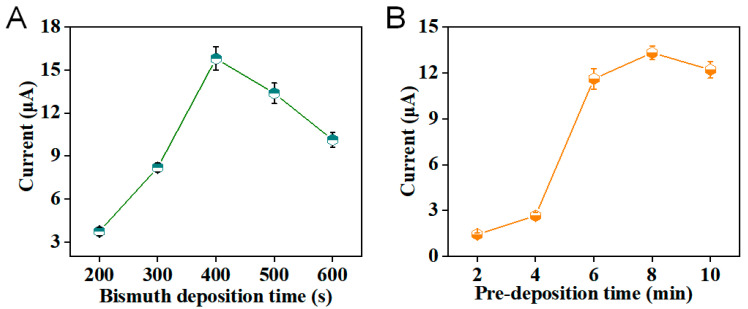
The optimization characterization of (**A**) the deposition time of bismuth (Bi) standard solution for the fabrication of Bi film in a sensing platform. (**B**) The pre-concentration time of Zn^2+^. The DPASV strategy was applied with hydrogels soaked in 1 μg/mL Zn^2+^ ABS buffer.

To realize the preferable electrochemical signals for the detection of sweat Zn^2+^ by DPASV, the deposition time of fabricated Bi film on the sensing electrode was surveyed. In Figure 3A, the optimal time was 400 s. Moreover, the electrochemical parameter of pre-deposition time for detecting Zn^2+^ by DPASV was investigated. As shown in Figure 3B, the optimal pre-deposition time was set at 8 min, achieving the highest current signal of Zn^2+^. All the optimization surveys were implemented by using the hydrogel-based flexible patch sensor after deposition of rGO at −1.2 V and Bi at −0.8 V on the electrode [18] by a PalmSense^®^ small electrochemical system with a bluetooth accessory (Houten, The Netherlands). The DPASV electrochemical technique was applied to detect 1 μg/mL Zn^2+^ solution.

### 3.3. rGO/Bi Film Flexible Patch Sensor for Zn^2+^ Sensing

**Figure 4:**
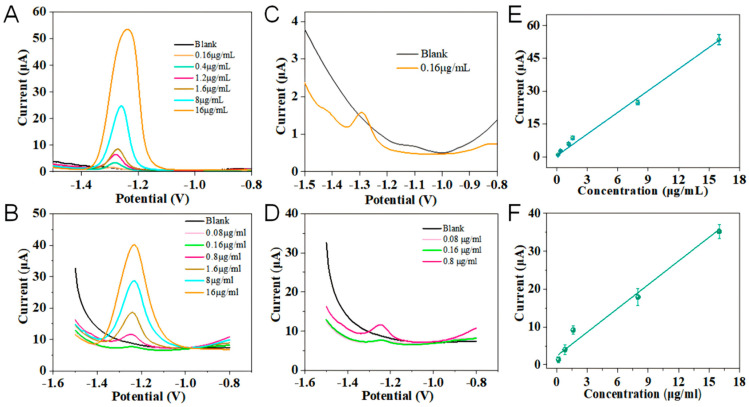
The concentration-dependent DPASV detection of Zn^2+^ using the flexible patch sensor installed with a hydrogel that soaked in 0.1 mol/L ABS buffer solution (**A**) and a hydrogel that soaked in artificial sweat (**B**). The zoomed-in low-concentration detection curves of Zn^2+^ in hydrogel with a 0.1 mol/L ABS buffer solution (**C**) and artificial sweat (**D**). The corresponding concentration linear dynamic plots of Zn^2+^ were obtained in the hydrogel with ABS buffer solution (**E**) and artificial sweat (**F**), respectively. The DPASV experimental conditions were as follows: pre-deposition voltage: −1.4 V; voltage ranged from −1.5 V to −0.8 V vs. Ag/AgCl.

Based on the above observation, the magnetic porous hydrogel used as a solid electrolyte and a sweat reservoir with a magnetically regulated structure has excellent electrochemical performance, implementing a fast and effective sweat collection. As indicated in Figure 4A,D, the fabricated hydrogel was used on the flexible patch sensor with a small magnet under the patch to fix the hydrogel and achieve the stable sensing platform, observing the concentration-dependent DPASV response to Zn^2+^ with the hydrogel containing the HAC-NaAC (pH 4.5) buffer (Figure 4A) and artificial sweat (Figure 4D). Figure 4B,E show the low-concentration signal curves of Zn^2+^. For each test, 4 μL Zn^2+^ solution with a certain concentration was added to the hydrogel. Clearly, Figure 4C,F show a similar wide linear relationship for detecting Zn^2+^, ranging from 0.16 µg/mL to 16 µg/mL with the hydrogel-based flexible patch sensor. The resulting current response depended on the Zn^2+^ concentration, following the regression equations of I_buffer_ = 0.3653 + 3.3097C (μg/mL), R^2^ = 0.9641 (n = 3), and I_sweat_ = 2.362 + 2.089C (μg/mL), R^2^ = 0.9641 (n = 3). The result shows that the prepared hydrogel-based flexible patch sensor for Zn^2+^ detection has the satisfied detection limit and could directly detect Zn^2+^ in sweat.

### 3.4. Interference

**Figure 5:**
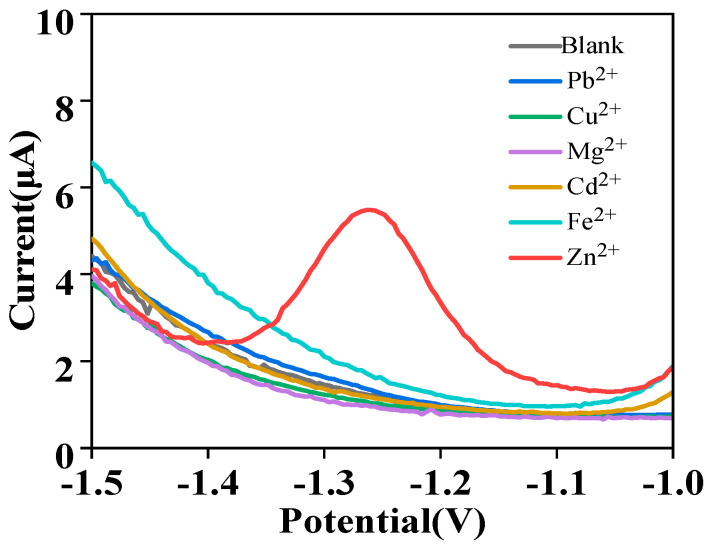
The interference for the flexible patch sensor was studied by detecting interfering ions with a concentration of 10 μg/mL, including Pb, Cu^2+^, Mg^2+^, Cd^2+^, Fe^2+^, and a mixture with 1 μg/mL Zn^2+^, respectively.

The possible interfering ions with a concentration of 10 µg/mL, such as Pb^2+^, Cu^2+^, Mg^2+^, Cd^2+^, and Fe^2+^, were studied by using a magnetic porous hydrogel-based flexible patch sensor. As shown in Figure 5, the hydrogel-based sensing platform exhibits good detection selectivity for Zn^2+^ at the concentration of 1 µg/mL. In all, the as-prepared hydrogel as a solid electrolyte possesses better electrochemical properties than liquid electrolytes. Because of the hydrogel having the arrangement of its inner structure regulated by an external magnet, the diffusion speed of Zn^2+^ ions to the surface of the electrode were enhanced, resulting in a favorable DPASV response.

### 3.5. On-Body Characterization of Flexible Zn^2+^ Patch Sensor

**Figure 6:**
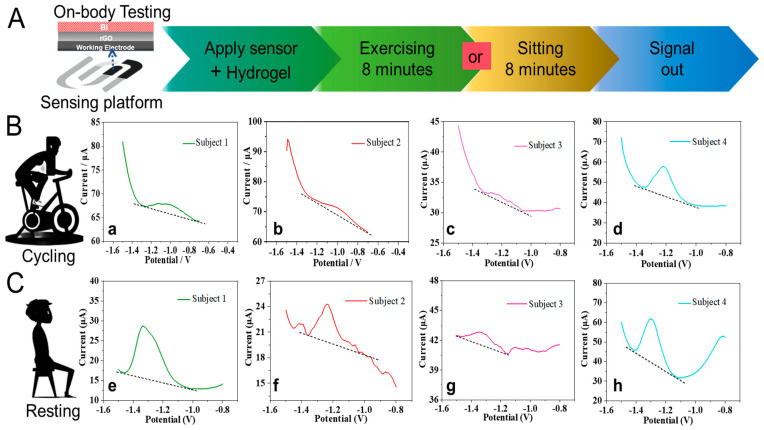
The strategy for sensing sweat Zn^2+^ on a hydrogel-based flexible patch sensor during routine activities (cycling and resting). (**A**) The modification and sensing process of on-body testing. (**B**) The flexible patch sensor for the detection of sweat Zn^2+^ of (a) male Subject 1, (b) male Subject 2, (c) male Subject 3, and (d) female Subject 4 during cycling. (**C**) The flexible patch sensor for the detection of sweat Zn^2+^ of (e) male Subject 1, (f) male Subject 2, (g) male Subject 3, and (h) female Subject 4 during sitting. The sweat Zn^2+^ on-body experimental conditions were as follows: pre-deposition voltage: −1.4 V; the voltage ranged from −1.5 V to −0.8 V vs. Ag/AgCl.

The on-body testing performance of the hydrogel-based flexible patch sensor was evaluated during routine activities (cycling and resting). The flexible patch sensor with hydrogel was stuck to the forearms of the participants (Figure 1D). In Figure 6A, the details of the on-body testing process were implemented by cycling (8 min) and resting (8 min), then recording the current signals. During the cycling test, the pre-concentration procedure was carried out along with the cycling for 8 min by volunteers. The prepared hydrogel with a porous structure could generate the electrochemical cell that established a fast sweat Zn^2+^ sensing platform. As shown in Figure 6B(a–d), the detection signals of Zn^2+^ from four subjects displayed stable background currents and stripping peaks. This indicates that the flexible patch sensor shows a reliable and stable sensing system even in cycling. In addition, the sweat Zn^2+^ of four subjects during the resting state has been investigated. As shown in Figure 6C(e–h), the four subjects keep sitting, and the flexible patch sensors stick to the subject’s forearm; then, using the DPASV method, pre-concentration was performed for 8 min, recording the sensing signals. Nevertheless, from the comparison of the recorded sensing signals between Figure 6B,C, the sensing signals of sweat Zn^2+^ at the resting state were higher than those in the cycling state. This is due to how sweating at rest involves secretion at much lower rates than during exercise [23,24], which to some extent leads to the different Zn^2+^ concentrations in hydrogel during exercising and sitting, as well as the sensing signals. As reported, low secretion rates of resting sweat and evaporation are challenging for sweat collection [23]. However, the proposed hydrogel-based sensing platform for monitoring Zn^2+^ shows the favorable sensing of signals during resting, indicating fast and effective sweat collection by hydrogel, leading to a rapid and accurate sweat Zn^2+^ sensing system.

Due to differences in daily diet, medication, and environmental effects, the sweat Zn^2+^ of subjects displayed signals at different levels. However, these results show well-defined Zn^2+^ stripping peaks around −1.3 V, indicating that the flexible Zn patch sensor can successfully detect the presence of Zn^2+^ in sweat during routine activities. In particular, the sensing strategy of natural sweat Zn^2+^ was successfully detected under a resting state, which could reflect more realistic fluctuations of Zn^2+^ in the subjects’ bodies [25,26], making it more user-friendly. In addition, the quantitative analysis of sweat Zn^2+^ was also studied by the standard additions of the collected sweat sample from Subject 3 (Figure S3) [11]. Consequently, the hydrogel-based flexible patch sensor displays good sensitivity and feasibility of application for sweat Zn^2+^ monitoring. 

## 4. Conclusions

In summary, we came up with an effective sensing strategy, for the first time using the fabricated magnetic porous hydrogel to collect sweat and generate the sweat reservoir on the sensing electrode to realize the detection of sweat Zn^2+^. The prepared hydrogel consists of magnetic Fe_3_O_4_ NPs and NH_2_-MWCNT to achieve an acceptable conductivity. Additionally, the CaCO_3_ as a pore-forming agent was added to hydrogel to generate the porous structure of hydrogel by acid etching. Notably, the hydrogel exhibits a homogeneous distribution of its inner structure due to the magnetic regulation of an external magnet during the curing process. Such hydrogel-based flexible patch sensors with good conductivity and absorptive properties were successfully applied to the on-body evaluation of sweat Zn^2+^ ions of four subjects. The uniform distribution of Fe_3_O_4_ NPs and porous hydrogels has increased absorption capability and electrolyte diffusion. As a result, the fabrication and implementation of the novel magnetic composite hydrogel as a solid electrolyte for sweat Zn^2+^ sensing has promising applications in real-time sweat monitoring. 

## Data Availability

Data will be made available upon request.

## Supplementary Materials

The following supporting information can be downloaded at https://www.mdpi.com/article/10.3390/s24175627/s1, Figure S1: the images of the flexible patch electrode; Figure S2: the synthetic details of the magnetic hydrogel; Figure S3: the quantitative analysis of sweat Zn^2+^ by the proposed method and ICP; Table S1: the comparable literature for the detection of sweat Zn^2+^ by wearable sensor.
