# Supplementary material for: Magnetic Porous Hydrogel-Enhanced Wearable Patch Sensor for Sweat Zinc Ion Monitoring
Source: Sensors (Basel). 2024 Aug 30;24(17):5627. doi: 10.3390/s24175627 (PMC11398112; doi:10.3390/s24175627)
Supplement: Supplementary file 1 [file sensors-24-05627-s001.zip › sensors-3172639-supplementary - proofreading - done.pdf]

Magnetic Porous Hydrogel-Enhanced Wearable Patch Sensor for Sweat Zinc ion Monitoring

Yao Chu †, Zhengzhong LvZeng †, Kaijie Lu, Yangyang Chen, Yichuan Shen, Kejia Jing, Haifeng Yang * and Wanxin Tang *

College of Chemistry and Materials Science, Shanghai Normal University, Shanghai 200234, China; 1000497336@smail.shnu.edu.cn (Y.C.); 1000526787@smail.shnu.edu.cn (Z.L.); 1000535232@smail.shnu.edu.cn (K.L.); 1000547249@small.shnu.edu.cn (Y.C.); 1000517384@smail.shnu.edu.cn (Y.S.); 1000535234@smail.shnu.edu.cn (K.J.)

* Correspondence: Correspondence: hfyang@shnu.edu.cn (H.Y.); wanxintang@shnu.edu.cn (W.T.)

† These authors contributed equally to this work.

Note S1. The Images of Flexible Patch Electrode

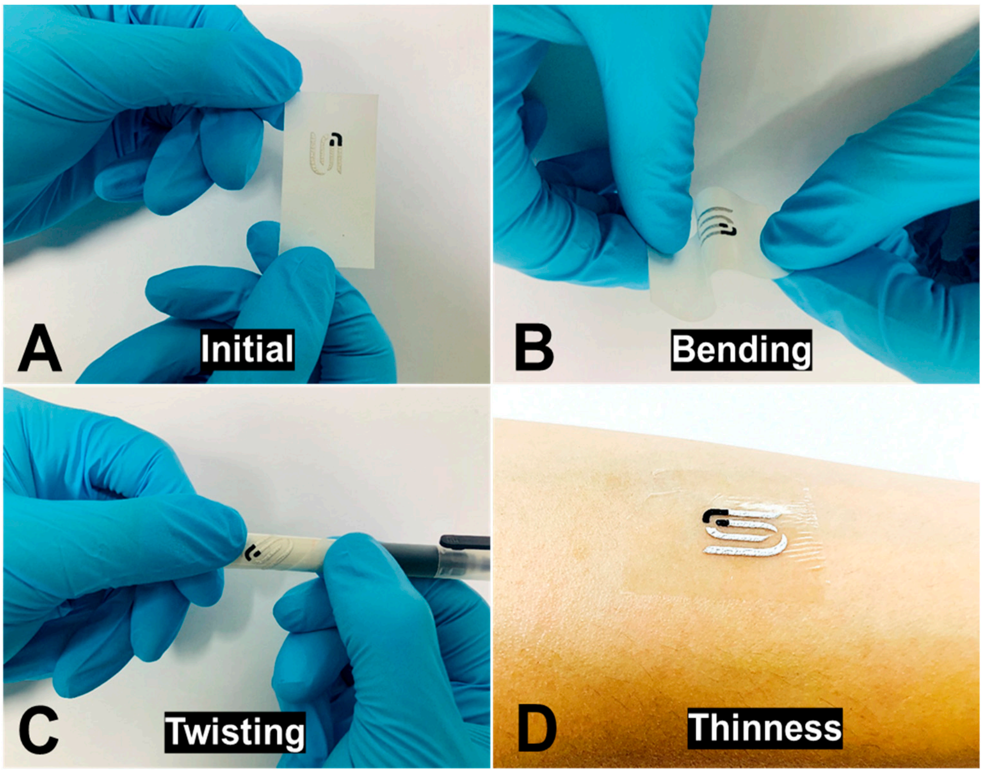

Figure S1. Images of flexible patch electrodes in different statuses. (A) Initial, (B) 90° bending, (C) twisting, and (D) the thinness and the good skin compactness.

Note S2. The Synthetic Details of Magnetic Hydrogel

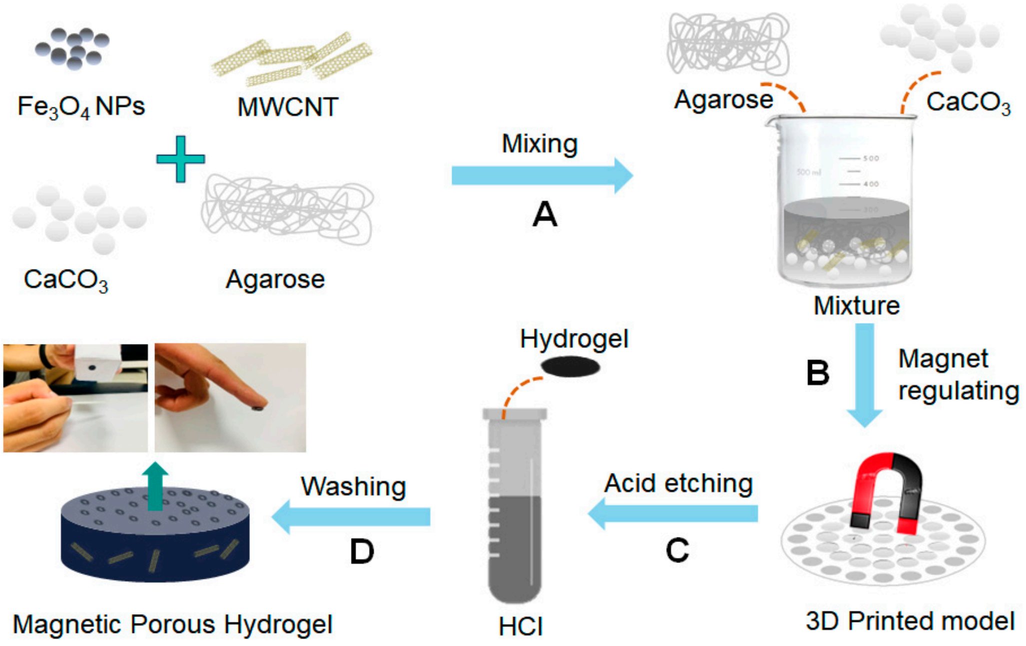

Figure S2. The synthetic process of magnetic hydrogel. (A) The fabricated materials of hydrogel were mixed with the DI water as the solvent. (B) A magnet regulated the hydrogel in a 3D-printed model during the curing process. (C) The cured hydrogels were etched with a hydrochloric acid (HCl) solution. (D) After the acid etching process, DI water-washed hydrogel to obtain the final magnetic porous hydrogel.

Note S3. The Quantitative Analysis of Sweat Zn^{2+} by the proposed method and ICP

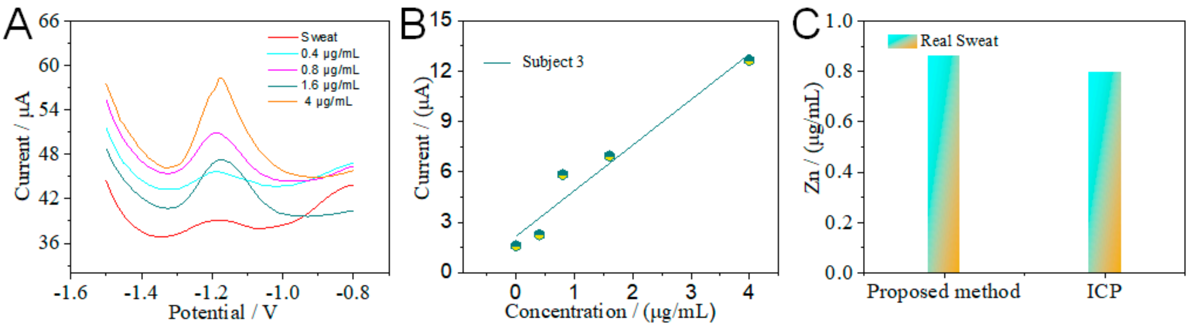

Figure S3. The quantitative analysis of sweat Zn^{2+}. (A) DPASV results for successive standard additions of Zn^{2+}, recorded with the flexible patch sensor in human sweat samples collected from subject 3. (B) The corresponding calibration plot of sweat Zn^{2+}. (C) The quantitative comparison of the sweat Zn^{2+} detected by the proposed method and inductively coupled plasma (ICP).

To quantify the amount of Zn^{2+} in sweat, the sweat of subject 3 was collected and analyzed. Due to the composition of sweat is complex, different for each individual. There are many affected factors could result in the change of sweat Zn^{2+}, the standard additions were applied for analysis of the collected real sweat sample (Figure S3). The corresponding linear standard addition plot with the regression equation of $I_{sweat3} = 2.713C(\mu g/mL) + 2.174$, R = 0.9747, and a zinc sweat concentration value of 0.8622 μg/ml, which is consistent with the reported range from 0.39 to 1.56 μg/mL [11], and compared to the ICP detection result in 0.7986 μg/mL, showing a certain relativity.

Note S4. Comparable Literature for Detection of Sweat Zn^{2+} by wearable sensor

Table S1. A summary of the reported wearable sensors for sweat Zn^{2+} detection

**Electrode**	**Technique**	**Detection limit (μg/mL)**	**Sensitivity （μA·ml/μg）**	**Sweating time (min)**	**Reference**
Tattoo paper	SWASV	0.05	23.8	15-20 min	[11]
Flexible patch	DPASV	0.0296	3.31	8 min	This work
Microsensor PET array	SWASV	—	10.4	15-20 min	[12]

As shown in Table S1, our proposed sensing platform has a short sweating time and favorable LOD. It displays the priority of fabricating the user-friendly wearable patch sensor and good sensing signals at rest sweat Zn^{2+} monitoring.
